# A Cross-Sectional Analysis of Community Water Fluoridation and Prevalence of Pediatric Dental Surgery Among Medicaid Enrollees

**DOI:** 10.1001/jamanetworkopen.2020.5882

**Published:** 2020-08-12

**Authors:** Helen H. Lee, Luis Faundez, Anthony T. LoSasso

**Affiliations:** 1 Department of Anesthesiology, University of Illinois at Chicago, Chicago; 2 Department of Economics, University of Illinois at Chicago, Chicago; 3 Department of Economics, DePaul University, Chicago, Illinois

## Abstract

**Importance:**

Dental surgery under general anesthesia (DGA) is an ineffective, costly treatment for caries. Interventions to reduce the need for DGA are challenging because children’s parents may not seek care until surgery is required. Community water fluoridation (CWF) effectively prevents early childhood caries, but its effectiveness in reducing severe early childhood caries is unknown.

**Objective:**

To determine whether access to CWF is associated with the prevalence of DGA.

**Design, Setting, and Participants:**

This is a cross-sectional analysis of Medicaid claims data from 2011 to 2012. Deidentified data were derived from Medicaid claims and enrollee files for Massachusetts, Texas, Connecticut, Illinois, and Florida for children aged 9 years and younger enrolled in either a fee-for-service or managed care plan through their state’s Medicaid program. Linear regression models tested for associations between CWF and covariates. Multivariable linear regression models tested for associations between CWF and outcomes. Regression models included clustered SEs at the county level. Data analysis was performed from December 2018 to March 2020.

**Exposures:**

Access to CWF was determined by estimating the proportion of a county’s total population that had access to a fluoridated public water system.

**Main Outcomes and Measures:**

The main outcome was county-level DGA prevalence. Other outcomes were caries-related visit prevalence and patient quality indicators (asthma and diabetes). Covariates included county-level demographic, socioeconomic, and dental practitioner variables.

**Results:**

A total of 436 counties within 5 states per year (872 county-year observations), were included in the analysis. Adjusted analysis revealed that a 10% increase in the proportion of county’s population access to CWF was associated with lower caries-related visit prevalence (−0.45 percentage points; 95% CI, −0.59 to −0.31 percentage points; *P* < .001). Increasing CWF access in 10% increments was associated with decreased DGA prevalence in unadjusted analysis (−0.39 percentage points; 95% CI, −0.67 to −0.12 percentage points; *P* = .006) but not in adjusted analysis (−0.23 percentage points; 95% CI, −0.49 to 0.02 percentage points; *P* = .07). Increasing the proportion of county’s access to CWF by 10% was not associated with the prevalence of asthma-related exacerbations (−0.02 percentage points; 95% CI, −0.10 to 0.05 percentage points; *P* = .53) or diabetes-related exacerbations (−0.0003 percentage points; 95% CI, −0.0014 to 0.0009 percentage points; *P* = .66).

**Conclusions and Relevance:**

This study extends our understanding of CWF’s benefits for children’s oral health. Specifically, these findings suggest that increasing a population’s access to CWF’s is associated with decreased caries-related visits and may also be associated with use of dental surgical services within high-risk populations.

## Key Points

**Question:**

Is access to community water fluoridation associated with lower rates of pediatric dental surgical procedures in high-risk populations?

**Findings:**

In this cross-sectional study of Medicaid-enrolled children from 5 states, increasing the proportion of the population exposed to community water fluoridation water was associated with a lower prevalence of caries-related visits in both adjusted and unadjusted analyses and with a lower prevalence of dental surgical procedures in unadjusted analysis only.

**Meaning:**

These findings suggest that for children at high risk of caries, community water fluoridation should be considered as an intervention to prevent childhood caries and to decrease the prevalence of dental surgical procedures.

## Teaser

This cross-sectional study of Medicaid-enrolled children from 5 US states evaluates whether access to community water fluoridation is associated with decreased prevalence of dental surgery under general anesthesia.

## Introduction

Community water fluoridation (CWF) is the most effective and cost- efficient dental public health intervention.^1^ Fluoridation of drinking water is among the top 10 US public health achievements of the 20th century.^2^ The use of CWF for population oral health has been associated with lower rates of caries, particularly in primary teeth.^3^ Regional studies^4,5^ have shown an association of CWF with reducing disease severity, which, in turn, is associated with utilization of caries-related procedures among residents aged 0 to 21 years. The timing of exposure to CWF is critical. Exposure in early childhood substantially reduces caries disease burden, not only during childhood but throughout the life span, with an exposure-response effect.^6^

However, the potential adverse outcomes of early childhood exposure to fluoridated water are controversial. Childhood exposure to fluoridated water has been associated with attention-deficit/hyperactivity disorder in the US.^7^ The recently described association of in utero exposure to fluoridated water with childhood neurodevelopmental issues^8^ is balanced by criticisms of study limitations and prior studies^9,10,11^ that refute the associations between fluoride and neurodevelopmental outcomes. Although the causal link between CWF and neurodevelopment and behavior disorders remains to be determined, fluoride’s benefits in preventing caries are better established.^12,13,14^ What is unknown is the extent to which CWF, an effective population-level preventive intervention, can reduce severe early childhood caries (S-ECC) and its associated treatments. The development of S-ECC is associated with a child’s social determinants of oral health, which reflect factors related to the child’s environment and the family’s oral health beliefs, behaviors, and parent-child personal dynamics.^15^ Provision of general anesthesia has been recognized as a necessary service to facilitate dental treatments in patients who require extensive treatment, have special health care needs, and/or experience acute situational anxiety,^16,17^ which is often the case in children with S-ECC. In the US, dental operations performed under general anesthesia (DGAs) represent a significant financial burden to public insurance programs such as Medicaid, which provides health care coverage for low-income children and adults, with state-level variations in eligibility.^18,19^ Under the Early and Periodic Screening, Diagnosis, and Treatment Program, Medicaid covers DGA events for select children. Therefore, DGA events do not pose great financial stress to families. However, on the health care system level, Medicaid expenditures on pediatric hospital and ambulatory surgery center–based DGA have been estimated to be approximately $450 million.^18^ Total Medicaid expenditures are likely much higher, because office*-*based DGAs account for 40% to 75% of all DGA events in some states.^19^ Furthermore, provision of general anesthesia to young children has been scrutinized because of a 2015 US Food and Drug Administration warning on the use of commonly used anesthetic agents,^20^ which may not be supported in the translation from basic science to clinical trials.^21,22,23,24,25,26,27^ The objective of this study is to determine whether access to CWF is associated with the prevalence of DGA events among young Medicaid-enrolled children across 5 states.

## Methods

### Study Design and Setting

In this cross-sectional analysis of Medicaid claims data from 2011 to 2012, the unit of analysis is on the county level, which was defined using the county of residence variable in the Medicaid enrollment file. This study was approved by the institutional review board of the University of Illinois at Chicago. Because the study used claims for health services used by Medicaid-enrolled children across several states, obtaining informed consent was not feasible; therefore, a waiver of informed consent was granted. This study follows the Strengthening the Reporting of Observational Studies in Epidemiology (STROBE) reporting guideline for cross-sectional studies.^28^

### Study Population and Setting

The definition of S-ECC refers to clinical disease in primary dentition among children younger than 6 years.^29^ State Medicaid programs vary in their coverage of general anesthesia for dental surgery, with an upper limit defined up to age 8 years.^30,31^ We included children aged 9 years or younger enrolled in either a fee-for-service or managed care plan through their state Medicaid program in 2011 to 2012. The unit of analysis was on the county level, and prevalences of outcomes were based on claims for services.

### Data

A convenience sample of deidentified data was derived from Medicaid claims and enrollee files for Massachusetts, Texas, Connecticut, Illinois, and Florida in 2011 to 2012. Specifically, Medicaid Analytic eXtract *Personal Summary* and *Other Therapy* files were obtained from the Centers for Medicare & Medicaid Services.

### Explanatory Variable

We defined county-level access to CWF by creating a continuous variable to estimate the proportion of a county’s population with access to fluoridated water (proportion CWF) with values 0 to 1 representing 0% to 100% of a county’s population. Centers for Disease Control and Prevention data on fluoridation of public water systems (PWSs) is found on the My Water’s Fluoride website.^32^ The Centers for Disease Control and Prevention treats a PWS as fluoridated if the fluoride concentration is 0.6 mg/L (parts per million) or greater. The proportion of a county’s access to CWF was estimated as follows:
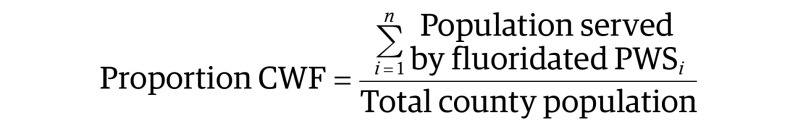
,where *i* denotes the number of PWSs.

### Outcomes: Surgical Events, Caries-Related Visits, and Patient Quality Indicators

The primary outcome of interest was receipt of caries-related surgical treatment with general anesthesia, or DGA. Individuals diagnosed with dental caries were identified using the *International Classification of Diseases, Ninth Revision, Clinical Modification*, codes 521.00 through 521.09.^33^ The provision of general anesthesia was identified with the following American Dental Association’s *Code on Dental Procedures and Nomenclature* and/or the American Medical Association’s *Current Procedural Terminology* codes: D9220, 00170, 00172, 00174, and 00176. Caries-related dental procedures were identified with the following *Code on Dental Procedures and Nomenclature* codes: D0120, D0150, D0210, D0272, D0330, D1120, D1203, D1351, D2150, D2331, D2930, D3220, D3310, and D7140.^34^ The prevalence of DGA was calculated as the proportion of children who had a DGA event among children who had a caries-related visit.

Caries-related visit prevalence was a secondary outcome. As a more general measure of population disease burden, the prevalence of caries-related visits was estimated by the proportion of children with a caries-related claims (defined earlier in this article) among all children enrolled in Medicaid in the study period.

We defined patient quality indicators as secondary outcomes. The Agency for Healthcare Research and Quality (AHRQ) has created Prevention Quality Indicators (PQIs) to be used as tools to identify hospital admissions for conditions that should be treated and managed on an outpatient basis. PQIs are used to identify ambulatory care–sensitive conditions, which provide “insight into the quality of the health care system outside the hospital setting.”^35^ The PQI for asthma and diabetes admission rates is defined by AHRQ as discharges with a principal *International Classification of Diseases, Ninth Revision, Clinical Modification* code for asthma (49300-49302, 49310-49312, 49320-49322, 49381, and 49390-49392) or diabetes (25010-25013, 25020-25023, and 25030-25033). PQIs for asthma exclude cases with diagnosis codes for cystic fibrosis and anomalies of the respiratory system. We limited PQIs to children aged 0 to 9 years to remain consistent with age limitations of caries-related visits and DGA visits.

### Covariates

The primary threat to the validity of our findings is that of omitted variable bias—in other words, any association between CWF and DGA might be due to confounding factors that affect both DGA and CWF levels. For example, if low-income counties tend to have low access to fluoridation, failure to control for income levels could lead to biased results. All covariates were estimated at the county level to compare with county-level estimates of outcomes. Variables included county-level demographic characteristics, such as age (proportion of population <10 years old), race/ethnicity (White, Black, or Hispanic), and measures of socioeconomic status. To measure county-level socioeconomic status, the enrollee county data from Medicaid Analytic eXtract Enrollment files were linked to the Health Resources and Services Administration’s Area Resource File.^36^ County-level socioeconomic status variables included percentage of persons born outside the US, per capita personal income, median household income, percentage of persons in deep poverty (income <50% of the federal poverty level),^37^ percentage of persons in poverty (income <100% of federal poverty level), percentage of persons aged 25 years and older with education less than a high school diploma, percentage of persons aged 25 years and older with 4 or more years of college, unemployment rate, and median home value. Because a greater supply of dentists is associated with improved oral health outcomes among children,^38^ we included the ratio of dentists per 100 000 people on a county level. We display our regression results with and without covariates to test the sensitivity of our findings to potential omitted variable bias.

### Statistical Analysis

We measured associations between CWF and covariates using linear regression models. Multivariable linear regression models tested for associations with caries-related visit prevalence, DGA prevalence, and PQIs (asthma and diabetes). Regression models included clustered SEs at the county level. We used 2-sided *t* tests to determine statistical significance, which was determined a priori to be *P* < .05. Counties with missing data for covariates (8 counties over the course of 2 years) were not included in analysis (eTable 1 in the Supplement). Imputation of the missing data using state-level means was performed and included in a sensitivity analysis (eTable 2 in the Supplement). Data management and analysis was performed using STATA statistical software version 14.2 (StataCorp). Data analysis was performed from December 2018 to March 2020.

## Results

A total of 436 counties per year across 5 states were included in the analysis, yielding 872 county-year observations. The unit of analysis is on the county level. The mean proportion of a county’s population with CWF access was 0.69 (95% CI, 0.67-0.71) (Table 1). The mean prevalence of caries-related visits was 0.150 (95% CI, 0.145-0.155) and that of DGA visits (surgical visits among caries-related visits) was 0.10 (95% CI, 0.09-0.11).

**Table 1.** County-Level Sociodemographic, Dental Workforce, and Oral Health Characteristics for Medicaid-Enrolled Children in 5 States, 2011-2012

County-level characteristics	Value, mean (SD)
Proportion with community water fluoridation, mean (95% CI)^a^	0.69 (0.67-0.71)
Caries-related visit prevalence, mean (95% CI)	0.150 (0.145-0.155)
Dental surgery under general anesthesia prevalence, mean (95% CI)^b^	0.10 (0.09-0.11)
Race/ethnicity, %	
Non-Hispanic	
White	0.68 (0.214)
Black	0.07 (0.076)
Hispanic	0.22 (0.219)
Persons born outside the US, %	8.1 (7.2)
Income, $	
Per capita personal	37 674 (9063)
Median household	45 119 (10 578)
Persons in deep poverty, %^c^	6.8 (2.9)
Persons in poverty, %^d^	17.3 (5.9)
Persons aged ≥25 y with education less than high school, %	17.1 (7.9)
Persons aged ≥25 y with ≥4 y college, %	20.0 (8.8)
Unemployment rate, %	7.8 (2.2)
Median home value, $	120 218 (76 832)
Dentists per 100 000 population, No.	33.0 (22.3)
Total population	150 868 (464 335)
Population aged <10 y, %	12.8 (2.43)
Observations, No.^e^	872 (872)

^a^ Proportion of county population with access to community fluoridated water.

^b^ Calculated as the proportion of children with caries-related visits who had a dental surgery under general anesthesia visit.

^c^ Deep poverty is defined as income less than 50% of the federal poverty level.

^d^ Poverty is defined as income less than or equal to 100% of the federal poverty level.

^e^ A total of 436 counties across 5 states were included for 2011 to 2012, resulting in 872 county-year observations.

Increasing the proportion of a county’s access to CWF was associated with decreased caries-related visits. Every 10% increase in the proportion of the population’s access to CWF was associated with a decrease in the prevalence of caries-related visits in both unadjusted analysis (−0.31 percentage points; 95% CI, −0.47 to −0.15 percentage points; *P* < .001) and adjusted analyses (−0.45 percentage points; 95% CI, −0.59 to −0.31 percentage points; *P* < .001) (Table 2).

**Table 2.** Association Between Proportion of County Population With Access to Community Water Fluoridation and Pediatric Oral Health Outcomes

Characteristics of regression model	Prevalence of caries-related visits		Prevalence of DGA visits^a^	
	Unadjusted	Adjusted	Unadjusted	Adjusted
Proportion with community water fluoridation (range, 0-1), percentage points, mean (95% CI)^b^	–0.31 (–0.47 to –0.15)	–0.45 (–0.59 to –0.31)	–0.39 (–0.67 to –0.12)	–0.23 (–0.49 to 0.02)
*P* value	<.001	<.001	.006	.07
Observations, No.	872	872	872	872
Demographic controls^c^	No	Yes	No	Yes

Abbreviation: DGA, dental surgery under general anesthesia.

^a^ Calculated as the proportion of children with caries-related visits who had a DGA visit.

^b^ Proportion of county population with access to community fluoridated water, ranging from 0%-100% (0-1).

^c^ Demographic controls include percentage born outside the US, per capita personal income, median household income, percentage of persons in deep poverty (defined as income <50% of the federal poverty level), percentage of persons in poverty (defined as income <100% of federal poverty level), percentage of persons aged 25 years and older with less than a high school diploma, percentage of persons aged 25 years and older with 4 or more years college, unemployment rate, median home value, total county population, number of dentists per 100 000 population, fraction Black non-Hispanic, fraction Hispanic, and percentage of county population younger than 10 years.

The prevalence of dental surgery ranged from 6% to 14% and generally decreased as the proportion of the county’s population access to CWF increased from 0% to 100% (Figure). Increasing CWF access in 10% increments was associated with decreased DGA prevalence in unadjusted analysis (−0.39 percentage points; 95% CI, −0.67 to −0.12 percentage points; *P* = .006) but not in adjusted analysis (−0.23 percentage points; 95% CI, −0.49 to 0.02 percentage points; *P* = .07) (Table 2).

**Figure.**
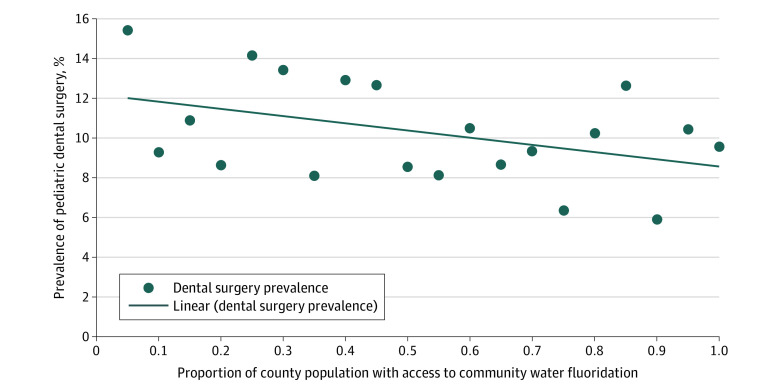
Decreasing Dental Surgery Prevalence With Increasing Proportion of Population With Access to Community Water Fluoridation Graph depicts the association of dental surgery prevalence with proportion of population with access to community water fluoridation. Proportion of county population with access to community water fluoridation ranges from 0% to 100% (0-1).

To test whether the association of CWF access with surgical prevalence could be explained by other factors regarding utilization of health services (eg, poor access to timely preventive services or characteristics of the population that might be related to preventive and tertiary health service utilization), we tested for the association between differences in access to CWF and exacerbations of asthma and diabetes (Table 3). In adjusted analysis, a 10% increase in the proportion of a county’s access to CWF was not associated with asthma exacerbations (mean dependent variable, 0.0749; −0.02 percentage points; 95% CI, −0.10 to 0.05 percentage points; *P* = .53). Similarly, adjusted analysis revealed no association between increasing the proportion of county’s access to CWF by 10% and diabetes exacerbations (mean dependent variable, 0.00015; −0.0003 percentage points; 95% CI, −0.0014 to 0.0009 percentage points; *P* = .66).

**Table 3.** Association Between Counties With Proportion of County With Access to Community Water Fluoridation and Pediatric Ambulatory Care Quality Indicators

Characteristics of regression model	Asthma	Diabetes
Proportion with community water fluoridation, percentage points, mean (95% CI)^a^	−0.02 (−0.01 to 0.05)	−0.0003 (−0.0014 to 0.0009)
*P* value	.53	.66
Mean dependent variable	0.0749	0.00015
County-level demographic controls^b^	Yes	Yes
Observations	872	872

^a^ Proportion of county population with access to community fluoridated water, ranging from 0%-100% (0-1). Exacerbations of asthma and diabetes are considered preventable with access to preventive medical care, per Agency for Healthcare Research and Quality’s Pediatric Quality Indicator, among children aged 0 to 9 years.

^b^ County-level demographic controls include percentage born outside the US, per capita personal income, median household income, percentage of persons in deep poverty (defined as income <50% of the federal poverty level), percentage of persons in poverty (defined as income <100% of federal poverty level), percentage of persons aged 25 years and older with less than high school diploma, percentage of persons aged 25 years and older with 4 or more years college, unemployment rate, median home value, total county population, number of dentists per 100 000 population, fraction Black non-Hispanic, fraction Hispanic, and percentage of county population younger than 10 years.

Sensitivity analysis to test the effect of missing data on the association between CWF access and primary outcomes yielded no change in coefficient magnitude or significance of association compared with analysis that excluded counties with missing data. In adjusted analysis, increasing the proportion of a county’s access to CWF by 10% was associated with a decrease in caries-related visits by 0.427 percentage points (95% CI, −0.566 to −0.289 percentage points; *P* < .001) but was not associated with a decrease in DGA visits (−0.236 percentage points; 95% CI, −0.496 to 0.025 percentage points; *P* = .08) (eTable 2 in the Supplement).

## Discussion

This study supports prior work on the benefits of CWF beyond primary prevention.^14^ The financial burden of pediatric DGA in hospital and ambulatory settings on the Medicaid system has been estimated to total $450 million.^18^ However, we have observed that a large proportion of DGA events occur in dental office settings in select states, suggesting that the true total financial burden to the Medicaid system exceeds $450 million.^19^ We found that, in unadjusted analysis, increasing a population’s access to CWF was associated with lower DGA prevalence among children who had caries-related visits, which may serve as a proxy for S-ECC. In addition, our findings provide a greater perspective on our understanding of risks and benefits associated with children’s exposure to fluoride. Although recent work suggested that there is an association of in utero exposure to fluoride with early childhood neurodevelopment,^8^ it should be noted that criticism has been aimed at multiple study limitations (eg, nonhomogeneous distribution of data, potential errors and biases in the estimation of material fluoride exposure and in outcome measurement, and potential omitted variable bias).^11^ Furthermore, prior work has not supported an association between fluoride exposure and pediatric neurodevelopment.^9,10^ Our findings, as they relate to tertiary oral health services, are unexpected, because the development of S-ECC in the US is largely thought to be driven by oral health behaviors, such as preventive dental care, regular toothbrushing, or reducing intake of sugary foods and beverages,^39,40,41,42^ which have all been targeted by interventions to improve children’s oral health.

Because of concerns regarding potential omitted variable bias, we estimated regression models with outcomes that are unlikely to be directly associated with fluoridation levels but would instead reflect unmeasured county-level socioeconomic characteristics. Specifically, we wanted to determine whether CWF is associated with higher admission rates for another preventable health condition, such as exacerbations associated with asthma or diabetes. Again, PQIs would be associated with CWF only if other (unobserved) local area factors related to the health care system and socioeconomic determinants of health were associated with CWF levels. Finding an association between CWF and PQIs would tend to invalidate any observed association between CWF and DGA because it would instead suggest the presence of confounding variables (ie, omitted variable bias). The lack of an association between CWF and PQIs suggests that an association between CWF and DGA is unlikely to be related to unobserved local area factors.

In addition to reducing severe caries, the use of CWF may help avoid a preventable surgery with general anesthesia, which has multiple benefits. A preponderance of basic science and animal model studies^43,44,45,46^ have demonstrated the neurotoxic and neurological effects of commonly used medications for moderate sedation and general anesthesia. Ongoing clinical trials^21,25^ have not supported an association between single anesthetic exposure and general intelligence or learning, but there is evidence suggesting that multiple exposures may decrease neuropsychological domains affecting executive functioning.^25,47^ The US Food and Drug Administration approved a label change regarding possible neurotoxic effects of general anesthetic and sedation medications in children younger than 3 years, warning that “exposure to these medicines for lengthy periods of time or over multiple surgeries or procedures may negatively affect brain development in children younger than 3 years.”^20^ Beyond potential adverse clinical outcomes, reducing dental surgical procedures has immediate financial implications for health care systems. Reducing the demand for dental operations addresses a substantial source of dental expenditures within the Medicaid system, because in a single-state study,^48^ 25% of dental expenditures were associated with 8% of children younger than 6 years. Furthermore, a child who presents for dental surgery is likely to require further treatment for caries in the future. Most patients who undergo DGA experience recurrence of dental disease within 12 to 24 months after surgery,^49,50^ likely because surgical interventions do not address the etiological factors rooted in behaviors and health beliefs. Primary prevention efforts are often aimed at changing oral health behaviors. However, among many families affected by S-ECC, oral health behaviors are associated with social determinants of health (eg, caregiver psychosocial factors, household financial insecurity, or prior negative experiences with the dental delivery system).^51^

### Strengths and Limitations

This study addresses possible confounding factors that might also explain the association between low CWF levels and higher DGA prevalence by adjusting for county-level demographics, socioeconomic indicators, and dental practitioner density. We also explored the possibility that counties with low CWF levels might also share characteristics with health care systems that experience high levels of pediatric asthma and diabetes exacerbations, which have been termed by the AHRQ as ambulatory care–sensitive indicators. Higher levels of these ambulatory care–sensitive indicators would signal that health care systems were not adequately addressing conditions that can be managed through access to timely preventive outpatient care.

Our study has a number of limitations to consider in the application of results to a more generalized population. First, data were sourced from 5 states. We did not include data from the noncontiguous states of Alaska and Hawaii, which are the 41st and 50th lowest states in terms of access to CWF.^52^ Geographical bias may have influenced our findings, because the data largely represent coastal areas of the US (Texas, Florida, Massachusetts, and Connecticut). Although our data included a wide range of DGA prevalence (<1% to >10%), inclusion of a greater number of states would minimize clustering effects by unmeasured sources. Data sources limited our ability to measure access to CWF among Medicaid-enrolled children. Our measure of county-level access to CWF represents the general county population and did not specify by insurance status. Data sources also limited our ability to determine the population’s use of PWSs. Although populations may have access to CWF, we were unable to verify the degree to which communities were consuming CWF or bottled water. Second, our interpretation of findings is limited by the cross-sectional study design, which allows us to comment on association, not causal inference, between CWF and DGA prevalence. Because of budget limitations, we were unable to obtain additional years of data, which would have allowed for longitudinal analysis. Third, our study design does not allow us to address an exposure-dose response. We did not assess the duration of time that counties had access to CWF, nor did we estimate the duration of time the individuals in our study population resided in counties with CWF.

## Conclusions

The adverse effects of fluoridated water have not been established beyond the level of association. Public health policy should be based on a stronger degree of certainty regarding cause and effect. The public health importance of our findings relates to the contribution toward the evidence of CWF’s benefits for children’s oral health, which is a well-established public health intervention for the primary prevention of caries. These findings suggest that among children who had caries-related visits, CWF may be associated with reduced development of S-ECC, as reflected by decreased use of surgical services in the unadjusted model. In addition, although access to CWF may be associated with lower pediatric dental surgery prevalence in the Medicaid population, it is important to note that dental surgical procedures persist in this population. Although broad policies may serve as an effective intervention to improve population oral health, this does not obviate the need for continued work to develop and test interventions that address oral health risk factors at the family level. Our findings should be incorporated into ongoing cost-benefit analyses of this public health intervention.

## Notes

Supplement. **eTable 1.** Comparison of Variables With and Without Missing Data, by County **eTable 2.** Association Between Proportion of County Population with Access to Community Water Fluoridation and Pediatric Oral Health Including Counties With Imputed Values for Missing Covariates
