# Supplementary material for: A Cross-Sectional Analysis of Community Water Fluoridation and Prevalence of Pediatric Dental Surgery Among Medicaid Enrollees
Source: JAMA Netw Open. 2020 Aug 12;3(8):e205882. doi: 10.1001/jamanetworkopen.2020.5882 (PMC7424407; doi:10.1001/jamanetworkopen.2020.5882)
Supplement: Supplement. — eTable 1. Comparison of Variables With and Without Missing Data, by County eTable 2. Association Between Proportion of County Population with Access to Community Water Fluoridation and Pediatric Oral Health Including Counties With Imputed Values for Missing Covariates [file jamanetwopen-3-e205882-s001.pdf]

Supplementary Online Content

Lee HH, Faundez L, LoSasso AT. A cross-sectional analysis of community water fluoridation and prevalence of pediatric dental surgery among Medicaid enrollees. *JAMA Netw Open*. 2020;3(8):e205882. doi:10.1001/jamanetworkopen.2020.5882

eTable 1. Comparison of Variables With and Without Missing Data, by County
eTable 2. Association Between Proportion of County Population with Access to Community Water Fluoridation and Pediatric Oral Health Including Counties With Imputed Values for Missing Covariates

This supplementary material has been provided by the authors to give readers additional information about their work.

© 2020 Lee HH et al. *JAMA Network Open.*

eTable 1. Comparison of Variables With and Without Missing Data, by County

	No Missing Data	Missing data	Difference
Variable			
**Caries-related visit prevalence**	0.1536	0.1776	-0.0240
**DGA prevalence**	0.1015	0.2078	-0.106***
**Proportion CWF**	0.6897	0.6080	0.0817
**% Foreign Born**	8.1	11.3	-3.2
**Per Capita Personal Income**	$ 37,675	$ 48,152	-10,477***
**Median Household Income**	$ 45,119	$ 45,537	418
**% Persons in Deep Poverty**	6.8	5.8	1.0
**% Persons in Poverty**	17.3	15.0	2.3
**% Persons 25+ with < High School Diploma**	17.1	21.9	-4.8*
**% Persons 25+ with 4+ years College**	20.0	18.3	1.7
**Unemployment Rate**	7.8	5.3	2.5***
**County-Year Observations**	872	16	888

Proportion CWF=proportion of county population with access to community water fluoridation, ranging from 0-100% (0-1). DGA=dental surgery under general anesthesia, calculated as the proportion of children with caries-related visits who had a DGA visit. A total of 436 counties were included in analysis and 8counties were excluded due to missing data. Over a two-year period, this represented 872 and 16 county-year observations. * p<0.05, ** p<0.01, *** p<0.001

© 2020 Lee HH et al. *JAMA Network Open.*

eTable 2. Association Between Proportion of County Population with Access to Community Water Fluoridation and Pediatric Oral Health Including Counties With Imputed Values for Missing Covariates

**Characteristics of regression model**	**Prevalence Caries-Related Visits**		**Prevalence of DGA Visits**	
	(unadjusted)	(adjusted)	(unadjusted)	(adjusted)
**Proportion CWF**[¥] (0-1)	-0.0287***	-0.0427***	-0.0403***	-0.0236*
**95% CI**	-0.0440, -0.0134	-0.0566, -0.0289	-0.0686, -0.0118	-0.0496, 0.0025
**p-value**	0.000	0.000	0.006	0.076
**County-year Observations**	888	888	888	888
**Demographic controls**	No	Yes	No	Yes

[¥]Proportion CWF=proportion of county population with access to community fluoridated water, ranging from 0-100% (0-1). DGA=dental surgery under general anesthesia, calculated as the proportion of children with caries-related visits who had a DGA visit. Robust standard errors (95% confidence intervals) clustered at the county level. Demographic controls include: Percentage Foreign Born, Per Capita Personal Income, Median Household Income, Percentage Persons in Deep Poverty, Percentage Persons in Poverty, Percentage Persons 25+ with < High School Diploma, Percentage Persons 25+ with 4+ years College, Unemployment Rate, Median Home Value, total county population, Number of Dentists per 100,000 population, Fraction Black Non-Hispanic, Fraction Hispanic, and Percentage of county population under 10 years old . * p<0.1, ** p<0.05, *** p<0.01

© 2020 Lee HH et al. *JAMA Network Open.*
